# Antibiotic Use in Beekeeping: Implications for Health and Environment from a One-Health Perspective

**DOI:** 10.3390/antibiotics14040359

**Published:** 2025-04-01

**Authors:** Michela Mosca, Andrea Gyorffy, Marcella Milito, Camilla Di Ruggiero, Alessandra De Carolis, Marco Pietropaoli, Luigi Giannetti, Francesco Necci, Francesca Marini, Daniele Smedile, Manuela Iurescia, Alessia Franco, Antonio Battisti, Pasquale Rombolà, Marcella Guarducci, Giovanni Formato

**Affiliations:** Istituto Zooprofilattico Sperimentale del Lazio e della Toscana “M. Aleandri”, 00178 Rome, Italy; michelamoscabologna@gmail.com (M.M.); andrea.gyorffy-esterno@izslt.it (A.G.); marcella.milito@izslt.it (M.M.); camilla.diruggiero@izslt.it (C.D.R.); marco.pietropaoli@izslt.it (M.P.); luigi.giannetti@izslt.it (L.G.); francesco.necci@izslt.it (F.N.); francesca.marini@izslt.it (F.M.); daniele.smedile-esterno@izslt.it (D.S.); manuela.iurescia@izslt.it (M.I.); pasquale.rombola@izslt.it (P.R.);

**Keywords:** environmental impact, oxytetracycline, honeybees, antimicrobial resistance (AMR), residues

## Abstract

Background: The use of antibiotics in beekeeping has potential implications for honeybee health and environmental contamination. Recent research indicates that extensive antibiotic use in beekeeping, especially oxytetracycline, promotes antimicrobial resistance in bee-related bacteria. Honeybees can transport oxytetracycline-resistance genes during foraging, potentially establishing reservoirs of resistance in the colony and facilitating intergeneric gene transfer among various gut bacteria as well as in the microbiome of the flowers and the wider environment, where honeybees can spread antibiotic-resistance genes over a large distance. This study investigates the effects of oxytetracycline hydrochloride (OTC) treatment on honeybees from a One Health perspective, examining antibiotic residues in honey, environmental spread, and the presence of tetracycline-resistance genes (TET-RGs). Methods: In the spring of 2022, two groups of four honeybee hives were placed near an almond grove in Central Italy. One group was treated with 1.68 g of OTC, while the other remained untreated. Samples were collected from bees, honey, hive entrances, and flowers before treatment and at 3 as well as 9 days post-treatment. OTC residues and TET-RGs were analyzed to assess contamination and resistance gene dissemination. Results: OTC residues were detected in honey from both treated (day 3: 263,250.0 ± 100,854.3 µg/kg; day 9: 132,600 ± 146,753.9 µg/kg) and untreated hives (day 3: 20.5 ± 8.2 µg/kg; day 9: 135.8 ± 198.6 µg/kg), suggesting cross-contamination. Residues were also found in almond tree flowers (0.7 ± 0.1 µg/kg), with TET-RGs (*tet*(K), *tet*(L), *tet*(M), *tet*(B), *tet*(O), *tet*(D)) detected pre- and post-treatment. In honeybee gut bacteria, resistance genes (*tet*(M), *tet*(A), *tet*(D), *tet*(B)) appeared post-treatment in both groups. No significant correlation was observed between hive distance and resistance gene presence in flowers, although the presence of other farms located within the bees’ flight range, in which OTC might have been used in the past, could have influenced the results. Conclusions: These findings highlight the risk of OTC-induced antibiotic cross-contamination and the spread of TET-RG, raising concerns for bee health and environmental safety. Given honeybees’ social nature and the negative effects of antibiotics on their health, an antibiotic-free management approach is recommended for sustainable apiculture.

## 1. Introduction

American foulbrood (AFB) and European foulbrood (EFB) are infectious honeybee diseases that might lead to the devastation of honeybee colonies. AFB is a notifiable disease in various countries and listed by the World Organisation of Animal Health [1], therefore posing an impediment to international trade. Even though the use of antibiotics is strictly regulated in the European Union (EU), several veterinary medical products (VMPs) containing antibiotics (ABs) as active ingredients (e.g., oxytetracycline hydrochloride (OTC), tylosin tartrate, sulfadimethoxine, and trimethoprim) are registered globally for use in honeybees and are authorized for the control of American foulbrood (AFB) and European foulbrood (EFB). Excessive application of antibiotics is most commonly reported in the control of AFB with veterinary medical products (VMPs) containing OTC [2], as well as in metaphylactic treatments. In the EU, OTC can only be used off-label for treating honeybees under the cascade principle [3,4]. After administrating ABs to the honeybees, AB residues can be detected not only in honey, but also in other bee-derived products such as royal jelly, propolis, and wax [5]. In humans, OTC residues consumed with food might cause allergic reactions, liver damage, dysbiosis, and gastro-intestinal disorders [6], and might accumulate in the developing bones and teeth [7]. OTC residues persist in the environment, posing both acute and chronic human and animal health risk, including the development of antimicrobial resistance (AMR), which is considered a major emergency globally [7,8,9,10].

In the Americas, *Paenibacillus larvae* (*P. larvae*) infection of the honeybee colonies has been controlled with OTC for decades, and as a result, widespread resistance to OTC has been reported in approximately 40% of Argentinian and 16% of North American *P. larvae* isolates [2]. In Uruguay and in Chile, where the use of OTC is not authorized for honeybees, no OTC-resistant *P. larvae* strains have been detected [11]. The US Food and Drug Administration (FDA) approved tylosin tartrate in 2005 and lincomycin hydrochloride in 2012 for the treatment of honeybees. Okamoto et al. (2021) detected tylosin- and/or lincomycin-resistant *P. larvae* strains in the USA in 2017 [2].

In case of humans, the most prominent carrier of pharmaceutical pollutants is food. Excretion following human or veterinary AB treatments with ABs is the primary route through which drugs enter the environment [9]. The expression of tetracycline-resistance genes can exert its effect through various pathways: energy-dependent efflux pumps—*tet*(A), *tet*(C), *tet*(G), *tet*(K), and *tet*(L); ribosomal protection proteins—*tet*(M), *tet*(O), *tet*(Q) and *tet*(W); and enzyme inactivation or modification—*tet*(X) [12,13,14]. While honeybees are reported to be sentinels for environmental pollution, including AB/AMR [15,16,17], and the possible effects of agricultural activities on honey production is widely investigated [18], to the authors’ knowledge, no scientific reports are currently available regarding the environmental impacts of ABs administered to honeybees. The present study aims to investigate the evolution of the impact of AB treatment in beekeeping, using a One Health approach. Specifically, it investigates the presence of tetracycline-resistance genes (TET-RGs) against oxytetracycline (OTC) in the intestinal microbiome of treated honeybees, as well as in the environmental microbiome exposed to the ABs through the bees (e.g., flowers, internal parts of the beehive). Additionally, the study will examine OTC residues in honey from both treated and untreated hives within the same apiary, and in flowers visited by treated honeybees.

## 2. Materials and Methods

### 2.1. Field Trial Setup

**Figure 1:**
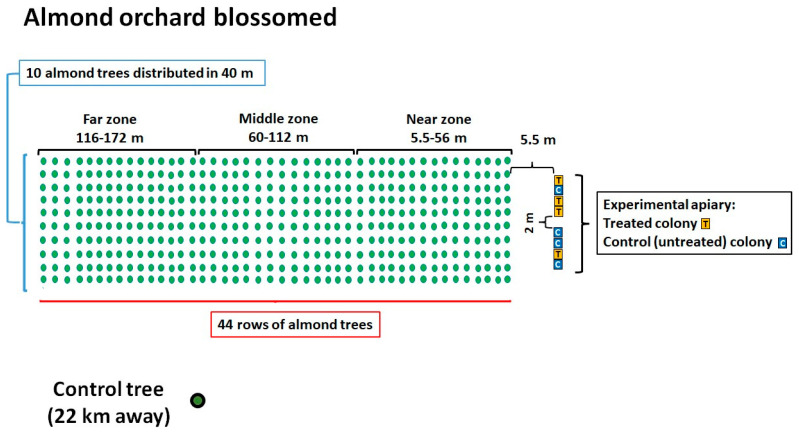
Diagram of the almond orchard showing the locations of the honeybee hives, their identification codes, and the experimental group (treated or control) to which they were assigned.

The trial was performed from February to March in 2022, to the east of Rome, Central Italy. Eight healthy colonies were placed in Dadant–Blatt ten-frame hives, in front of a blooming almond (*Prunus dulcis*) grove of 4040 m^2^ (Figure 1). The colonies were allotted to two groups, each consisting of four colonies (OTC-treated group and control untreated group). The colonies were homogenous concerning their strength, the number of frames covered by honeybees, and combs occupied by a brood. In all beehives selected for the trial, a laying queen was present along with 5–6 combs containing a capped and an uncapped brood, and 4–5 storage combs, all covered with adult bees for a total of 10 10-frame hives. The almond grove was selected because almond trees bloom the earliest in spring in Central Italy, and honeybees would focus their foraging activity on the almond blossoms located in front of the apiary. The orchard contained 440 trees arranged in 10 rows, with each row consisting of 44 trees.

**Figure 2:**
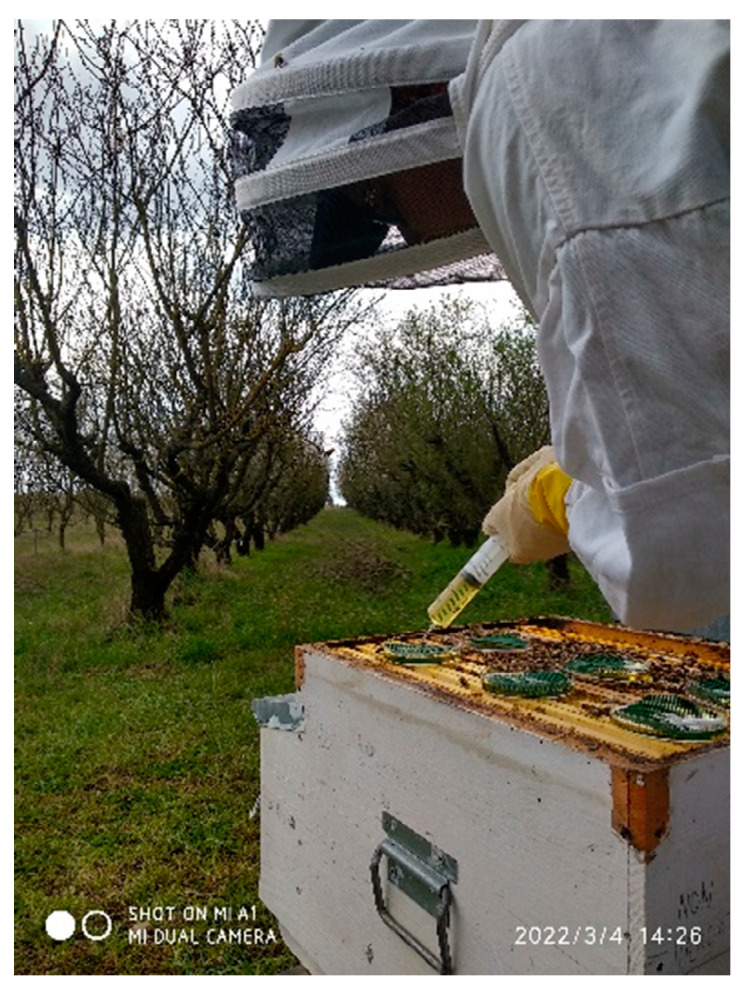
Administration of OTC to a hive in the treated group.

The almond orchard was divided into thirds based on their distance from the beehives: “near zone” (5.5–56 m distance), “middle zone” (60–112 m distance), and “far zone” (116–172 m distance) to determine whether there was a relationship between the potential presence of ABs in the flowers and their distance from the hive. A mature almond tree placed 22 km away from the grove served as the control. The 8 beehives were placed on 2 stands of 4 colonies each, which were 2 m apart from each other. The hives were placed about 5.5 m from the first line of almond trees (Figure 1). To evaluate the impact of the cross-contamination between OTC-treated and untreated colonies related to the position of the hives within the apiary, we identified two subgroups consisting of adjacent hives: subgroup 1, “one treated colony next to two control ones” (hive identification codes: K1, DB17, K2), and subgroup 2, “one untreated colony between two treated ones” (hive identification codes: DB11, 4OTC, DB4). The colonies of the treated group were administered with OTC (Zoetis Terramicin 100 (A.I.C. 100230034) per os) in a 1:1 diluted aqueous sucrose solution (Figure 2). Each colony in the treated group received 0.56 g of OTC orally, mixed in 180 mL of sucrose solution placed in Petri dishes, administered three times with a 5-day interval between treatments, following the protocol described by Mosca et al. (2022) [10]. The colonies of the control group received 180 mL of untreated (without OTC) syrup for each administration. The uptake of the treated and the untreated sucrose solution dosages were assessed after each administration. Samples were collected from adult honeybees, honey from the hive, dry swabs taken from the entrance of the bee hives, and flowers from the almond grove, both 7 days before the antibiotic treatment and 3 and 9 days after the last administration of OTC.

To assess the registered livestock farms within the foraging area of the honeybees located in the experimental apiary, as well as around the control tree, we collected geographical location data, the livestock species kept, and the start and end dates of farming activities for the livestock farms mandatorily registered in the National Animal Registry (“Banca Dati Nazionale,” BDN) [19]. These farms were located within a 3 km radius around the experimental apiary and a 1.5 km radius around the control tree. Only livestock farms that were active for at least one year between March 2012 and March 2022 were considered relevant, i.e., potential external sources of TET-RG contamination.

### 2.2. Chemical Analysis of the Samples

Honey samples were taken to assess OTC residues in honey of treated and untreated hives: a piece of uncapped honeycomb was taken from each colony nest in such a way that a sample of 5 cm × 4 cm was cut out from the upper part of the frames, right under Petri dishes, to represent the worst-case scenario. Since honeybees deposit honey from the top towards the bottom of the honeycomb, antibiotic concentration was expected to be higher in those cells [20]. Sampling was conducted in both experimental groups at three time points: before the OTC application, and 3 and 9 days after the last OTC treatment.

**Figure 3:**
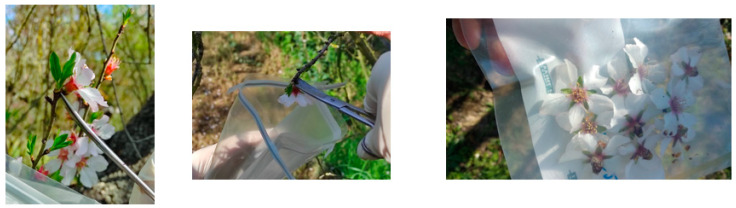
Sterile technique for collecting almond flower samples.

Almond flowers were collected using a sterile cut from the almond trees, ensuring that not only the petals, stamens, and pistils but also the receptacle was included from each flower. The staff involved wore disposable sterile gloves and FFP2 masks. The cut flower parts were allowed to fall directly from the branch into a sterile sampling bag (Figure 3). Between each sampling, the gloves were discarded, and the scissors were sterilized with a flame.

### 2.3. Assessment of OTC Residues

OTC residues were assessed both in honey as well as in the almond flowers.

To investigate the presence of OTC residues in honey and flowers, LC-MS/MS was applied. All reagents used for HPLC analysis were of analytical grade. The tetracycline standards of 99% purity degree were obtained from the Sigma-Aldrich Chemical Company (Darmstadt, Germany). Acetonitrile, methanol, and formic acid were provided by Carlo Erba Reagents (Cornaredo, Italy). Oasis HLB (60 mg, 3 mL) polymeric cartridges were purchased from Waters (Milford, MA, USA). Water was purified using the Milli-Q system (Merck, Darmstadt, Germany). Standard stock solutions (1 mg/mL) were prepared by weighing 10.0 ± 0.1 mg of standard substances and dissolving them in 10 mL of methanol using an amber glass. Working standard solutions in the mobile phase were prepared the day of analysis. An aliquot of 2 g of honey or 2 g of flower were mixed with 10 mL McIlvaine-EDTA for 15 min in an ultrasonic bath, and the extract was centrifuged. The centrifuged extract samples were loaded on an Oasis HLB (60 mg) SPE cartridge previously activated with 3.0 mL of methanol and 3.0 mL of deionized water, then the SPE cartridge was washed with a mixture of 3.0 mL of deionized water and 2.0 mL of methanol–water at 5:95 *v*/*v*. Tetracyclines were eluted with 3 mL of methanol containing 0.1% of formic acid, and the eluted liquid was evaporated at 40 °C under a nitrogen stream. The residue was finally dissolved in 0.5 mL of methanol–water at 50:50 *v*/*v* and injected into the LC-MS/MS system. Analyses were carried out with a QTRAP 5500 tandem mass spectrometer detector (AB Sciex, Framingham, MA, USA) equipped with a 1260 Infinity high-performance liquid chromatographer (Agilent Technologies, Santa Clara, CA, USA). The instrument was set in positive electrospray ionization mode with a capillary voltage of 5.5 kV and a source temperature of 500 °C. Ultra-pure air was used as a nebulizer gas, and ultra-pure nitrogen was applied as both a curtain and a collision gas. Positive ions were acquired in multiple reaction monitoring (MRM) mode, acquiring two or more diagnostic product ions from the chosen precursors to obtain high specificity and sensitivity. The chromatographic separation of the analytes was achieved on a Kinetex Biphenyl column (50 mm × 3.0 mm, 2.7 µm, Pnenomenex, Torrance, CA, USA) with a mobile phase of 0.1% formic acid in water (mobile phase A) and acetonitrile (mobile phase B) at a flow rate of 0.3 mL min^−1^, in gradient mode. The limit of detection (LOD) of the method is 0.1 µg/kg for tetracyclines and their epimers.

### 2.4. Bacterial Cultures, Identification, and Genetic Analysis

We collected one flower sample consisting of 50 individual flowers from each of these zones (near, middle, far) before as well as 3 and 9 days after the last OTC administration for a total of 9 samples. One sample was collected at the same 3 time points from the control tree, which was 22 km away from the apiary, resulting in 3 samples in total. To perform the investigation of OTC-resistance genes in bacterial strains, microbiological analyses were performed from sampled honeybees, dry swabs, and flowers.

Honey samples were taken to assess OTC residues in honey collected from both the treated and the untreated hives. A piece of uncapped honeycomb was taken from each colony nest in such a way that a sample of 5 cm × 4 cm was cut out from the upper part of the frames, right under Petri dishes [20]. Sampling was performed in both experimental groups at 3 time points: before the OTC application, and 3 as well as 9 days after the last OTC treatment.

Almond flowers were collected via a sterile cut from the almond trees in such a way that not only the petals, stamens, and pistils but also the receptacle was collected from each flower, and the staff involved was using disposable sterile gloves and FFP2 masks. The cut flower parts were allowed to fall from the branch directly into a sterile sampling bag (Figure 3). Between each sampling, the gloves were discarded and the scissors were sterilized with a flame.

From the beehives, worker bee and dry swab samples were collected. Concerning the honeybee samples, 10 live adult forager honeybees were collected from the lateral frames of the internal nest box of each colony. Sampling was performed before as well as 3 and 9 days after the last OTC administration, respectively, resulting in a total of 24 samples. Concerning the dry swab sampling, one dry swab sample was taken from the wooden entrance of each beehive, and afterward a mechanical cleaning was carried out by scraping with a sterile lever. The timing of the swabbing was 7 days before and 3 as well as 9 days after the last OTC treatment, with 24 dry swab samples collected altogether.

Table 1 shows the protocols used for bacterial culture isolation from flowers, swabs, and adult honeybees.

After triple smears on blood agar and Sabouraud dextrose agar, the blood agar plates were incubated under aerobic/anaerobic/microaerophilic conditions at 37 ± 2 °C for 24–48 h, while the Sabouraud dextrose agar plates were incubated at 37 °C for yeasts and at 25 °C for 5 days for molds.

After incubation, the bacterial colonies were identified based on their morphology, oxidase and catalase activity (using 3% hydrogen peroxide), Gram staining, and biochemical identification tests using API BioMérieux^®^ galleries. The results were determined by calculating the identification percentage and the typicality index of the species standards. The bacteria detected and identified were stored in Microbank^®^ and kept at −80 °C for any further identification for TET-RGs by molecular biological methods.

Out of 216 bacterial isolates identified biochemically, only bacteria that proved to be the most frequent (127 in total), based on their colony formation on the plates, were further analyzed for the detection of TET-RGs (16S rRNA amplicon; *tet*(K), *tet*(L), *tet*(M), *tet*(O), *tet*(A), *tet*(B), *tet*(C), *tet*(G), *tet*(D)) and for genus/species confirmation via PCR (Table 2).

### 2.5. PCR Assay to Investigate Tetracycline-Resistance Genes from Isolates

Genomic DNA extraction was performed using an automated system (QIAsymphony SP, Qiagen GmbH, Hilden, Germany) with the DSP Virus/Pathogen Mini Kit, following the manufacturer’s instructions. DNA concentration was determined using the NanoDrop Lite spectrophotometer (Thermo Fisher Scientific, Waltham, MA, USA). Extracted DNA was subjected to a nine-simplex PCR assay selected to investigate the presence of different TET-RGs in bacterial isolates obtained from almond flowers, and dry swabs taken from bee hives and worker bees. The resistance genes investigated included those coding the energy-dependent efflux protein pumps (*tet*(A), *tet*(B), *tet*(C), *tet*(D), *tet*(G), *tet*(K), *tet*(L)) as well as the genes coding the ribosomal protection proteins (*tet*(M), *tet*(O)) [22,23]. The amplification products were detected using the QIAxcel capillary electrophoresis (Qiagen GmbH, Hilden, Germany).

For a more accurate identification, the TET-RG-carrying isolates were identified by the 16S rRNA [24] Sanger sequencing, after PCR amplification of its 500 bp target region. The PCR products were purified using an enzymatic cleanup ExoSAP ITTM kit. Amplicons were Sanger sequenced on a 3500 Series Genetic Analyzer with BigDye Terminator chemistry (Applied Biosystems, Waltham, MA, USA) using the same primers. Sequence data analysis and trimming was performed using the CLC DNA workbench R software version 5.7.1.

### 2.6. Statistical Analysis

Data were analyzed using the XLSTAT™ software (Version 2023.1.6) [25], applying Wilcoxon’s signed-rank test with a two-tailed distribution and a two-sample *t*-test with a two-tailed distribution. Possible cross-contamination between the treated and untreated hives within subgroups 1 and 2 was analyzed by applying a simple repeated-measures ANOVA.

## 3. Results

### 3.1. Syrup Consumption and OTC Residues

Honeybees consumed all the solutions administered to each colony at each time point in both experimental groups.

**Figure 4:**
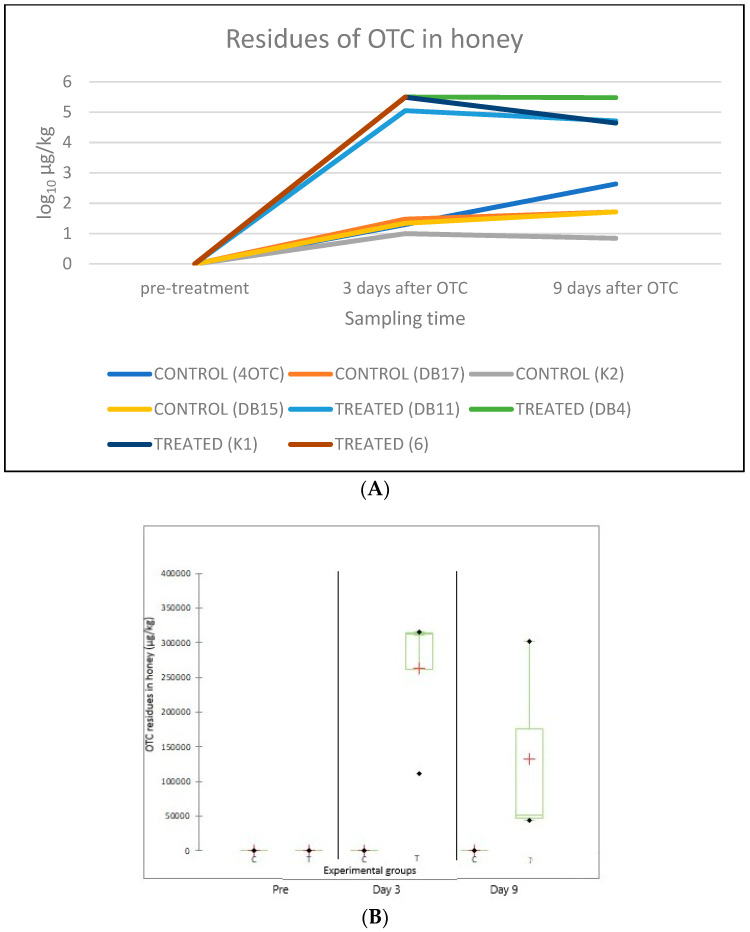
Amount of OTC residues (µg/kg) in honey samples from each beehive, taken before the OTC treatment (“pre-treatment”), and 3 and 9 days after treatment (**A**). The average amount of OTC residues (µg/kg, indicated by a red “+” sign) per experimental group (C: control; T: treated) before the OTC treatment (“Pre”), as well as on day 3 and day 9 after the treatment, is shown in panel. The bars represent the range of individual values while their standard deviation (SD) is also indicated (**B**).

Concerning the OTC residues in honey, all pre-treatment samples were negative (LOD: 0.1 µg/kg). After the OTC treatment, residues were detected in all the colonies in both the treated and the control groups. In the treated group, 3 days after the last antibiotic administration, the average amount of OTC residue was 263,250.0 ± 100,854.3 µg/kg, while 9 days after it was 132,600.0 ± 146,753.9 µg/kg. The average amount of OTC residue in the control group was 20.5 ± 8.2 µg/kg 3 days after the last OTC administration, while 9 days after the treatment, in the control groups it was 135.8 ± 198.6 µg/kg. A significant difference between the amount of OTC residue was detected 3 days after the last OTC administration between the treated and control groups C (*p* = 0.014). The position of the hives inside the apiary significantly (F-value: 0.002) affected the level of OTC residues (OTC contamination) (Table 3, Figure 4).

**Figure 5:**
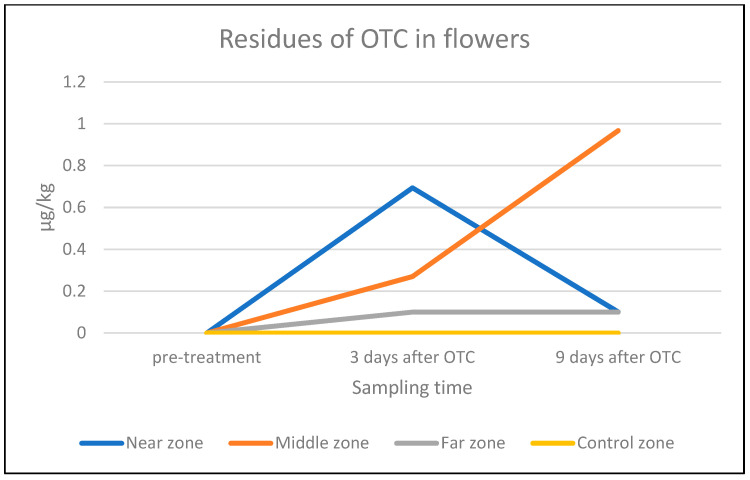
Average amount of OTC residue (µg/kg) in flowers from the near, middle, and far zones of the orchard and from the control tree (LOD = 0.1 µg/kg) on a log-10 scale.

Concerning the OTC residues in flowers, all flowers sampled before the administration of OTC were negative for OTC residues, including flowers originating from the control tree. Regarding flowers from the “far” zone, no OTC residues were detected either on day 3 or on day 9 after the last OTC administration. We detected OTC residues in the flowers sampled 3 days post-treatment from the “near” zone: 0.7 ± 0.1 µg/kg, and the “middle” zone: 0.3 ± 0.1 µg/kg. Nine days after the last antibiotic administration, no OTC was detected in the flowers from the “near” zone, while in the samples from the “middle” zone an average amount of 1.0 ± 0.1 µg/kg of OTC residues was found. No significant differences were found in the amount of OTC residues in flower samples from the different zones (Table 4, Figure 5).

**Figure 6:**
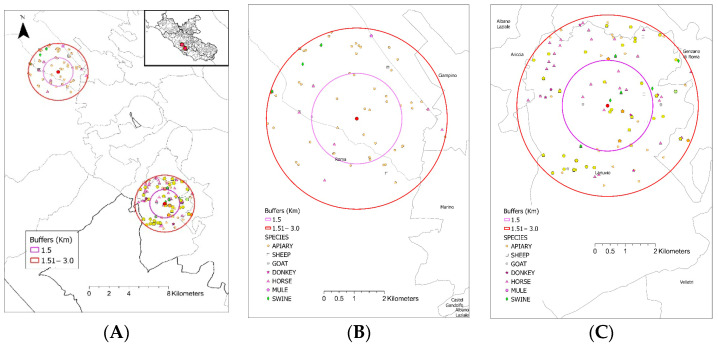
An overview of the location of the experimental apiary and the control tree and the registered livestock farms around. The experimental apiary is located at the bottom right, while the control tree is at the top left of the map (Panel (**A**)). Around each apiary, two buffer zones with radii of 1.5 km (purple line) and 3 km (red line) are shown. The registered livestock farms and livestock species kept within the buffer zones of the experimental apiary and the control tree are shown in panels (**B**) and (**C**), respectively.

Livestock farms within the 3 km radius of the experimental apiary that were active for at least one year during the period of March 2012–March 2022 (*n* = 210, out of which 21 were within a 1.5 km radius) according to the National Animal Registry were considered potential external sources of TET-RGs. The following livestock species were present in the selected livestock farms (in order of frequency: horse, honeybee, sheep, goat, swine, donkey, mule). Within the same radius and time period, a total of 201 farms surrounded the control tree (40 of which were within a 1.5 km radius), and some of these might have used tetracycline-class AB treatments (Figure 6).

### 3.2. Bacterial Strains and Genetic Analysis from the Different Sample Matrices

A total of 216 bacterial isolates were identified in the samples from the three matrices:

hives (next box), forager bees, and flowers. The 127 bacterial strains originating from all three types of matrices (66 from flowers, 27 from intestines, and 34 from dry swabs) identified as the most frequent were analyzed for TET-RGs (*tet*(K), *tet*(L), *tet*(M), *tet*(O), *tet*(A), *tet*(B), *tet*(C), *tet*(D), and *tet*(G). Out of the 127 bacterial strains selected, 114 were negative for TET-RGs, while the remaining 13 (10.2%) samples contained at least one TET-RG. These TET-RG-positive samples originated either from the germs isolated from flowers (9 samples, 7%), or from the ones isolated from the honeybee intestines (4 samples, 3%), while no bacterial strains isolated from the dry swabs were positive for TET-RGs.

Details of isolated strains from all three matrices containing TET-RGs are reported in Table 5.

#### 3.2.1. Honeybee Intestines

In the honeybee intestinal samples (altogether 24) taken before the OTC administration, no TET-RGs were detected in either group. Three days after the last antibiotic administration, seven TET-RGs against OTC were already detected: In the OTC-treated group, we identified 1 *tet*(M) gene in one *Bartonella apihabitans* isolate, as well as 2 *tet*(M) and 2 *tet*(A) genes in other two different isolates of *Bartonella apihabitans*, and one *tet*(D) in *Paenibacillus glucunolyticus*. In the control group, we detected 1 *tet*(B) gene in one *Bacillus cereus*. No TET-RGs were detected in the bacterial strains originating from the samples taken 9 days after the last OTC administration in either group (Table 5).

#### 3.2.2. Dry Swabs

No TET-RGs were found in bacterial strains isolated from the 24 dry swab samples taken at the entrance of the beehives, both pre- and post-treatment, in either group.

#### 3.2.3. Flowers

In the bacterial strains isolated from the 12 flower samples, 4 TET-RGs were identified before the OTC treatment: no such genes were found in the “near” zone; 1 *tet*(K) was found in *Staphylococcus equorum* and 1 *tet*(L) in *Alkalihalobacterium elongatum*, both in the “middle” zone; 1 *tet*(L) was found in *Chryseobacterium* spp., in the “far” zone; and 1 *tet*(M) was found in *Facklamia* spp. as well as one *tet*(B) in *Micrococcus* spp. in the control tree. Three days after the last OTC administration, one *tet*(O) was detected in a sample the “near” zone in *Cellulomonas*/*Microbacterium*.

Nine days post-treatment, one *tet*(D) was detected in *Kocuria varians* in the “near” zone and one *tet*(B) in *Micrococcus* spp. in the “far” zone. While 3 days after the last OTC treatment no TET-RGs were detected in the flower samples originating from the control tree, nine days after the last OTC administration one *tet*(K) was identified in *Kocuria* spp..

## 4. Discussion

### 4.1. OTC Residues in Honey

In all honey samples taken from each colony of the OTC-treated group, residues were detected both 3 (263,250.0 ± 100,854.3 µg/kg) and 9 days (132,600.0 ± 146,753.9 µg/kg) after the last antibiotic administration. In the treated group, the mean value of OTC residues decreased to half between post-treatment day 3 and 9. Thompson et al. (2007) [26] treated honeybee colonies with OTC and described very high levels (3700 µg/kg) of OTC residues even 8 weeks after the OTC application. The half-life of OTC residues in honey is 13 days when OTC is applied in 200–250 mL of liquid sucrose, and the time required for OTC residues to decline below the detection limits depends on both the half-life and the initial amount of residue [27]. OTC residues were also found in all colonies of the untreated (control) group, and the average amount of OTC residue increased by more than 6 times over the monitoring period, from 20.5 ± 8.2 µg/kg on day 3 to 135.8 ± 198.6 µg/kg on day 9 after the last OTC administration. The fast reduction in the OTC residue level in the treated group might have been caused by various factors: the intensification of the nectar collection by bees in parallel with the advancing of the spring season. The honey super withholding period (treatment period + label withdrawal time) set by the Food and Drug Administration (FDA) for the veterinary medicinal product applied is 54–57 days, considering factors such as the expansion of the colonies, honey consumption by adult bees (especially those feeding the larvae), the drift of honeybees carrying the antibiotic from treated beehives to untreated ones (drift behavior), and the transfer of OTC from the bee hives to the environment as a result of their foraging activity.

The present data underline the significance of cross-contamination that might occur among treated and untreated beehives, similar to what we observed in our previous study [10]. Regarding cross-contamination between treated and untreated beehives based on the spatial positioning of the colonies, the highest amount of OTC residue (432.0 µg/kg) in the control group, 9 days after the last OTC treatment, was found in the untreated colony situated between two treated colonies, representing the worst-case scenario in terms of placement. In this case, the likelihood of double cross-contamination was high due to the drifting of adult bees from both neighboring treated colonies on either side. The statistical analysis showed that the position of the hives inside the apiary during springtime significantly (F-value: 0.002) affected the level of OTC residues in the untreated colonies.

Regarding environmental pollution by ABs due to the treatment of farmed animals near beehives, scientific reports in the literature already exist, highlighting the potential impact within the foraging range of bees (typically 1–3 km). Savarino et al. (2020) [18] observed that antibiotics and their metabolites might contaminate the environment not only directly from the excretes of livestock, but also indirectly, through plants that absorbed drug metabolites from the soil. Tetracycline is poorly absorbed by mammalians; therefore, 30–90% of the drug administered per os is excreted in the form of its active metabolites both in the urine and in the feces of the treated animals [28,29,30], leading to increased antimicrobial contamination of the environment [31,32]. Tetracycline residues have been detected in irrigation water (140 ppb), pig waste lagoons (700 ppb), soil (25,000 ppb), hospital effluents (530 ppb), and wastewater treatment plants (920 ppb) [33]. Among others, the presence of tetracyclines as well as tylosin have been demonstrated in plant products (lettuce, carrots, tomatoes, maize) intended for human consumption [34].

### 4.2. OTC Residues in Flowers

Nowak et al. (2021) [21] demonstrated a relationship among the presence of *Lactobacilli* in flower pollen and those found in honeybee intestines. In the present study, we aimed at pioneering the investigation of whether AB administration to honeybees could result in environmental pollution, especially in flowers due to the specific biology of honeybees. In the present study, we demonstrated for the first time that honeybees treated with OTC could contaminate almond flowers, albeit at low levels, during their foraging activity.

No OTC residues were found in the almond flower samples collected before the administration of OTC nor in the control tree flowers. On the contrary, 3 days after the last antibiotic administration, OTC residues were found in flowers from the zone “near” the apiary (0.7 ± 0.1 µg/kg) and from the zone located at a “middle” distance from the apiary (0.3 ± 0.1 µg/kg). In the flowers collected from the “middle” zone, OTC residues were also detected (1.0 ± 0.1 µg/kg) 9 days after the antibiotic administration. Since the colonies were not artificially fed after the OTC treatment, the bees might have been more actively searching for foraging sources with the advancement of time [26]. Foraging honeybees need fully blooming flowers to collect nectar and pollen. The *Prunus dulcis* tree blossoms in general between February and March for 3–4 weeks, depending on the microclimatic conditions [35]. As the bloom progressed and the number of flowers decreased, the reduced flower availability may have resulted in higher concentrations of OTC residues per flower, attracting relatively more honeybees compared to the full bloom period of the orchard. Christiano et al. (2010) [36] reported that OTC is thermostable on fruit tree leaves but is rapidly degraded by the radiation of the sun (44% degradation in 1 day and 92% degradation in 4 days) as well as by rainfall (a 2-min-long simulated rainfall decreased the amount of OTC residues by 67%). Therefore, the amount of residue found on given days might also have been influenced by the actual meteorological conditions. In the “far” zone, no OTC residues were found in flowers collected, which could be attributed to the dispersal effects in the environment of the bees.

No significant differences were found in the amount of OTC residues in flower samples originating from the “near” and “middle” distance zones. The presence of very low OTC residues dispersed in the environment could pose a significant risk for the development of antimicrobial resistance (AMR), particularly due to the selection pressure exerted by the environment. Water is considered the primary source of residues contributing to AMR [37,38].

### 4.3. Bacterial Strains Isolated from Honeybee Intestinal Samples

After the administration of OTC, a reduction in *Lactobacillus* spp. isolates was observed in the honeybees of the OTC-treated group, along with an increase in environmental bacteria in the intestines. These environmental bacteria were not detectable in the intestinal samples collected before the antibiotic treatment, likely due to their low concentration at that time. *Corynebacterium* spp. and *Bacillus* spp. *Bartonella apihabitans* were not isolated from the bee intestines in either the control and or the treatment group before OTC treatment. *Bartonella apihabitans* was described and characterized in honeybee hind-gut samples [39]. In general, *Bartonella* spp. is prevalent in the guts of wintering bees and might be in relation to colony survival. *Bartonella* spp. was also detected in the *Varroa destructor* samples originating from the winter hive debris [39]. Further studies are needed to explore the role of *Bartonella apihabitans* in the transmission of resistance genes following AB treatments in bees. OTC treatment may have favored non-resistant but dominant (or subdominant) bacterial species that carry (multiple) TET-RGs, allowing them to better compete with the intestinal microbiota after being exposed to antibiotic selection pressure.

Long-term OTC treatment causes the accumulation of TET-RGs in the bacterial microbiome of honeybees. In the USA, where OTC is authorized and used for AFB and EFB, *tet*(B), *tet*(C), *tet*(D), *tet*(H), *tet*(L), *tet*(Y), *tet*(M), and *tet*(W) were detected in the intestinal bacteria of honeybees, often at high frequencies [2,13,27]. In samples originating from countries like EU Member States that do not allow the treatment of honeybees with antibiotics, the incidence of TET-RGs is considerably lower. Bacterial strains isolated from the intestines of honeybees in Switzerland, the Czech Republic, and New Zealand contained *tet*(B), *tet*(C), or *tet*(W), but lacked *tet*(D), *tet*(H), *tet*(Y), *tet*(M), and *tet*(L), similar to the TET-RGs identified in the microbiota of wild bumblebees in Connecticut, USA [13].

In commercial honey samples originating from the US, strains of tetracycline-resistant *P. larvae* expressing *tet*(L) in plasmids were isolated [40]. The *tet*(L) gene on plasmid pMA67 is responsible for most, if not all, of the tetracycline resistance detected in *P. larvae* in North America [41]. The spread of TET-RGs typically occurs through horizontal gene transfer of mobile genetic elements such as phages and/or plasmids [42]. Natural ecosystems and gut microbiota are common environments where horizontal gene transfer takes place among diverse bacterial species and their strains [40], which reinforces the evidence that antibiotic treatments, even when appropriately administered, might increase the prevalence of antimicrobial resistance (AMR).

In our study, in the bee intestinal samples taken before the OTC administration, no TET-RGs were detected in either group. Three days after the last antibiotic administration, seven TET-RGs were detected: In the OTC-treated group, we identified one *tet*(M) gene in *Bartonella apihabitans* as well as two *tet*(M) and 2 *tet*(A) genes in two other *Bartonella apihabitans* isolates, and one *tet*(D) in a *Paenibacillus glucunolyticus* isolate. One *tet*(B) gene was detected in *Bacillus* spp. from an untreated beehive. To the best of our knowledge, we have demonstrated for the first time that *Bartonella apihabitans* can carry TET-RGs. In another previous study of ours, *tet*(A), *tet*(M), *tet*(O), *tet*(B), and *tet*(D) were found in the honeybees sampled after the OTC treatment [10]. The *tet*(L) gene, which is typical in North America, where OTC treatment is approved for honeybees, was also detected in our field trial, highlighting the negative impact of OTC application in honeybee colonies due to antimicrobial resistance (AMR).

*Bacillus cereus* strains isolated from honey samples carried a variety of TET-RGs, including *tet*(L), proving that the *Bacillus cereus* in honey could act as a reservoir for TET-RGs [43]. In the intestinal samples, we detected a strain of *Bacillus* spp. carrying one *tet*(B). *Gilliamella apicola* and *Snodgrassella alvi* are reported to be highly resistant to tetracycline related to *tet*(B), *tet*(C), and *tet*(H). *tet*(B) is reported to be ubiquitous, occurring also in the sludge of sewage treatment plants, fish farming ponds, surface waters, and swine lagoons [17]. Since we detected mammalian pathogen bacterial strains in each matrix we investigated in the current study (e.g., *Staphylococcus caprae* in the pre-treatment dry swab samples, *Klebsiella pneumoniae* in the pre-treatment honeybee intestinal samples), it cannot be excluded that animal breeding activities took place within the nectar and pollen foraging range of the honeybees, and those establishments may have also served as potential sources of TET-RGs originating from livestock treated with OTC. In past decades, the entire study area in Central Italy has been extensively used for sheep breeding. Within 10 years of the onset of the experiments, a significant number of livestock farms remained within the foraging area (3 km radius) of the experimental apiary, housing various livestock species, including swine and cattle, which are more likely to undergo antimicrobial treatments [44]. Moreover, the apiary of IZSLT, where OTC treatment was documented one year before the onset of our present experiment, might have also served as a source of TET-RGs. In the buffer zone with a radius of 1.5 km, only apiaries were active during the examined period, with the exception of one horse farm. More information about the eventual antibiotic treatments in the neighboring livestock farms is warranted for the examination of antimicrobial residues and resistance on the environmental and farm levels.

Nine days after the last OTC treatment, we could not identify any TET-RGs in the bee intestinal samples. Bacterial-resistance genes impose a fitness cost on bacteria and might also be modified by other, concurrent genetic modulations [45,46].

### 4.4. Genetic Analysis in Strains from Dry Swabs

No TET-RGs were detected in the bacterial strains isolated from dry swabs taken at the entrance of the beehives.

The bacterial strains and changes in them detected in the dry swab samples followed the pattern already observed in the honeybee intestinal samples, including the presence of different *Lactobacillus* spp. After the OTC treatment, the *Serratia* opportunistic facultative pathogen species also appeared.

### 4.5. Genetic Analysis and Bacterial Strains Isolated from Flowers

In the bacterial strains isolated from the flower samples, four TET-RGs were identified before the OTC treatment: no such genes were found in the “near” zone; one *tet*(K) was found in *Staphylococcus equorum* and one *tet*(L) in *Alkalihalobacterium elongatum*, both in the “middle” zone; one *tet*(L) was found in *Chryseobacterium* spp., in the “far” zone; and one *tet*(M) was found in *Facklamia* spp., as well as one *tet*(B) in *Micrococcus* spp. in the control tree. Three days after the last OTC administration, one *tet*(O) was detected in a sample the “near” zone in *Cellulomonas*/*Microbacterium*.

Nine days post-treatment, one *tet*(D) was detected in *Kocuria varians* in the “near” zone, and one *tet*(B) in *Micrococcus* spp. in the “far” zone. While 3 days after the last OTC treatment, no TET-RGs were detected in the flower samples originating from the control tree, nine days after the last OTC administration, one *tet*(K) was identified in *Kocuria* spp.

TET-RGs detected in the flower samples were different from those identified in the honeybee intestinal samples and were linked to different bacterial strains. Therefore, flowers can be passive reservoirs of TET-RGs while having no impact on the TET-RGs in honeybee intestinal bacteria, and vice versa.

It is hypothesized that TET-RGs might also be of flower origin, associated with *Lactobacillales* and *Fructilactobacillus* [17]. Six TET-RGs were identified in the flowers sampled over the course of the trial, one *tet*(M), two *tet*(K), two *tet*(L), and one *tet*(O). The honeybees during their foraging activity could passively transport bacteria carrying TET-RGs from flowers to the colony. The transfer of the *tet*(L)-encoding plasmids between *P. larvae* strains is possible, as is an intergeneric transfer from other Gram-positive bacteria, such as *Barghavaea*, *Sporosarcina*, *Lactobacillus*, and the ubiquitous *Bacillus* spp. [40]. TET-RGs in colony microbiota may act as reservoirs of tetracycline resistance [47]. Many bacterial species, including *Bacillus* spp. and *Paenibacillus* spp., are known to persist in honey [40,48,49,50]. Honey is a potential source of TET-RGs for *P. larvae* [2]. Isolated *Bacillus cereus* strains were reported to carry various tetracycline-resistance genes, including *tet(L)* and *tet(K)*, from honey samples, highlighting the potential role of bacteria in honey as reservoirs for TET-RGs [43]. In this regard, it is reassuring that the *tet*(L) detected in flowers was not carried by bacteria responsible for the intergeneric transfer of that TET-RG but rather by *Alkalihalobacterium elongatum* and *Chryseobacterium* spp.

It was reported that 22.5% of the honeybees’ bacterial flora could also be detected in the mixed flower samples collected close to the apiaries, which might also pave the way to sharing TET-RGs between foraging honeybees and flowers in the foraging area of an apiary [51].

The microbiome of the flowers was the most variable among the three matrices, which might have been due to the exposure of the flowers to all environmental factors, among which are passive (e.g., the wind) and active (e.g., wasps) carriers of TET-RG-contaminated particles.

The influence of environmental-resistance genes, potentially originating from farms surrounding apiaries with a history of tetracycline use, should be considered. This is supported by recent scientific literature [17,52].

## 5. Conclusions

The present field trial is the first evidence that the administration of OTC to treat honeybees resulted not only in the cross-contamination of the honey in the brood chamber but also in spreading in the environment (flowers) surrounding the apiary. Our results also reported how cross-contamination between treated and untreated hives may depend on the position of the untreated hives respect to the treated ones. The possibility of such cross-contamination with OTC was forecasted by our previous study [10]. Cross-contamination between treated and untreated hives was reported in the case of different miticides, with tau-fluvalinate resulting in the most intense example of this phenomenon [53]. Even though amount of residue was mostly low in all cross-contaminated matrices, this does not reduce the risks of developing AMR. Low amounts of antibiotic residue in the environment (e.g., in flowers) might be responsible for the transmission of TET-RGs through bacteria of human, livestock, wildlife, and plant origin. In soil bacteria, various fertilizers are able to highly impact the environmental spread of tetracycline-resistance genes [17]. Albeit environmental contamination linked with honeybee treatments is to a much lower extent, it does not prevent the risk of AMR being developed. Honeybees forage usually up to 3 km around the beehive, potentially bringing and taking drug residues at low dosage and ARGs in the environment, including livestock farms, plants, and other apiaries [18,54] that might also have been present in the case of our current experiment based on the distance of registered, active livestock farms and the species kept around the experimental apiary. Within the beehive, the spread of TET-RGs might appear not only because of antibiotic residues, but also due to bacteria that potentially carry TET-RGs, through social interaction (e.g., trophallaxis), via consumption of bee bread and the stored honey, or through direct contact among bees and with the parts of the hive (wall surface, comb material) [17,21,55] (Nowak et al., 2021; Avila et al., 2023; Resci and Cilia, 2023). However, the study was conducted on the most frequently isolated bacteria from the different matrices. Further studies are warranted for investigating even more strains of bacteria. Our results adopting the One Health approach highlighted that antibiotics should be completely avoided in a sustainable beekeeping management. Proper hive management with the adoption of best beekeeping practices, including biosecurity measures, should completely prevent and eliminate the use of antibiotics in beekeeping, reaching antibiotic-free status for modern, sustainable apiculture. Due to the specific peculiarities of bees as social insects, due to the inefficacy of the use of antibiotics against infectious honeybee pathogens, and due to the risk of developing AMR, beekeeping should be considered an antibiotic-free sector.

## Figures and Tables

**Table 1:** Protocols used for bacterial culture isolation from flowers, swabs, and adult honeybees.

Matrix	Protocol
Flowers	Collect 50 almond flowers in a sterile, sealed bag. Add 100 mL of sterile physiological solution (1:3 dilution) and homogenize for 2 min. Streak the homogenate onto three blood agar plates and one Sabouraud agar plate using a 10 µL loop.
Swabs	Swab the hive walls and place the swab in a 16 mL Falcon tube with 10 mL of sterile physiological solution. Vortex for 1 min. Transfer the swab onto three blood agar plates and one Sabouraud agar plate using sterile forceps. Perform triple smears: first directly from the swab, then using a 10 µL loop [10].
Honeybees	Collect 10 live adult bees from each hive in a sterile bag. Extract intestines under sterile conditions and transfer them to a 16 mL test tube. Add 1 mL of sterile physiological solution and homogenize with a sterile swab. Perform triple smears using a 10 µL loop onto three blood agar plates and one Sabouraud agar plate [10,21].

**Table 2:** Number of bacterial isolates identified across the different matrices versus the number of isolates processed for TET-RG investigation by PCR.

	Pre-Treatment Sampling	3 Days After the Treatment	9 Days After the Treatment	Total
Flowers	32/31	33/12	17/23	82/66
Swabs	16/11	14/6	19/12	49/29
Intestines	18/3	39/20	28/2	85/27
Total	66/45	86/40	64/37	216/127

**Table 3:** Position of the beehives and OTC residues in honey.

OTC Treatment	Beehive Identification Code	OTC Residue Level Before the OTC Treatment (µg/kg)	OTC Residue Level 3 Days After the Last OTC Treatment (µg/kg)	OTC Residue Level 9 Days After the Last OTC Treatment (µg/kg)
Treated (subgroup 2)	DB11	Below LOD	112,000	51,700
Control (subgroup 2)	4OTC	Below LOD	20	432
Treated (subgroup 2)	DB4	Below LOD	316,000	302,000
Treated (subgroup 1)	K1	Below LOD	311,000	44,100
Control (subgroup 1)	DB17	Below LOD	30	52
Control (subgroup 1)	K2	Below LOD	10	7
Treated	6	Below LOD	314,000	No honey in the supers
Control	DB15	Below LOD	22	52

**Table 4:** Average amount of OTC residue (µg/kg) in the flower samples taken from the near, middle, and far zones of the orchard as well as from the control tree (LOD = 0.1 µg/kg).

Zone	Pre-Treatment (22 February 2022)	3 Days After the Treatment (16 March 2022) (µg/kg)	9 Days After the Treatment (24 March 2022) (µg/kg)
Near	Below LOD	0.7 ± 0.1	Below LOD
Middle	Below LOD	0.3 ± 0.1	1.0 ± 0.1
Far	Below LOD	Below LOD	Below LOD
Control tree	Below LOD	Below LOD	Below LOD

**Table 5:** TET-RGs identified in the respective bacterial strains isolated from the three different matrices (almond flower samples, hive boxes, and adult honeybees), according to their categorization (for flowers: near/middle/far zones and control tree; for hive boxes and honeybees: treated and untreated (control) groups) before the OTC treatment as well as 3 and 9 days after it, respectively.

Matrix	Categorization Within the Matrix	Pre-Treatment	3 Days After OTC Treatment	9 Days After OTC Treatment
Almond flowers	Near zone	N/A	1 *tet*(O) in *Cellulomonas*/*Microbacterium*	1 *tet*(D) in *Kocuria varians*
	Middle zone	1 *tet*(K) in *Staphylococcus equorum* 1 *tet*(L) in *Alkalihalobacterium elongatum*	N/A	N/A
	Far zone	1 *tet*(L) in *Chryseobacterium* spp.	N/A	1 *tet*(B) in *Micrococcus* spp.
	Control tree	1 *tet*(M) in *Facklamia* spp. 1 *tet*(B) in *Micrococcus* spp.	N/A	1 *tet*(K) in *Kocuria* spp.
Hive boxes	Treated group	N/A	N/A	N/A
	Untreated group	N/A	N/A	N/A
Honeybees	Treated group	N/A	1 *tet*(M) in *Bartonella apihabitans* 2 *tet*(M) and 2 *tet*(A) in *Bartonella apihabitans* 1 *tet*(D) in *Paenibacillus glucunolyticus*	N/A
	Untreated group	N/A	1 *tet*(B) in *Bacillus cereus*	N/A

## Data Availability

All data are available at IZSLT on request.
